# Indeterministic Data Collection in UAV-Assisted Wide and Sparse Wireless Sensor Network

**DOI:** 10.3390/s24196496

**Published:** 2024-10-09

**Authors:** Yu Du, Jianjun Hao, Zijing Chen, Yijun Guo

**Affiliations:** 1 Business School, Beijing Language and Culture University, Beijing 100083, China; duyu@blcu.edu.cn; 2 Beijing Key Laboratory of Network System Architecture and Convergence, Beijing University of Posts and Telecommunications, Beijing 100876, China; jjhao@bupt.edu.cn (J.H.); chenzijing@bupt.edu.cn (Z.C.)

**Keywords:** trajectory planning, indeterministic data collection, wireless sensor network, internet of things

## Abstract

The widespread adoption of Internet of Things (IoT) applications has driven the demand for obtaining sensor data. Using unmanned aerial vehicles (UAVs) to collect sensor data is an effective means in scenarios with no ground communication facilities. In this paper, we innovatively consider an indeterministic data collection task in a UAV-assisted wide and sparse wireless sensor network, where the wireless sensor nodes (SNs) obtain effective data randomly, and the UAV has no pre-knowledge about which sensor has effective data. The UAV trajectories, SN serve scheduling and UAV-SN association are jointly optimized to maximize the amount of collected effective sensing data. We model the optimization problem and address the indeterministic effective indicator by introducing an effectiveness probability prediction model. The reformulated problem remains challenging to solve due to the number of constraints varying with the variable, i.e., the serve scheduling strategy. To tackle this issue, we propose a two-layer modified knapsack algorithm, within which a feasibility problem is resolved iteratively to find the optimal packing strategy. Numerical results demonstrate that the proposed scheme has remarkable advantages in the sum of effective data blocks, reducing the completion time for collecting the same ratio of effective data by nearly 30%.

## 1. Introduction

### 1.1. Background and Motivation

With the rapid development of the Internet of Things (IoT), wireless sensor networks (WSNs) have been widely applied in many consumer electronics applications, such as intelligent transportation, forest monitoring, smart farms, smart ocean, E-commerce, environmental monitoring and emergency rescue [1,2]. Many studies have been conducted in this field. Refs. [3,4] studied the physical layer techniques, Refs. [5,6,7] focused on optimizing the network efficiency, Refs. [8,9] tackled the problem of network security, and [10,11] researched on the analysis and mining of sensing data. A basic and key problem faced by these smart applications is acquiring sensing data from sensor nodes timely and effectively. It is predicted that the number of sensors in the world is expected to exceed 100 trillion by 2030 [12]. Hence, in WSNs, how to collect a huge amount of sensor data while satisfying the low-energy consumption, low delay, and high reliability requirements of IoT applications is a challenging problem.

In many IoT applications, wireless sensor nodes (SNs) have been deployed in remote and harsh environments [13], where it is inconvenient to deploy ground infrastructure to collect sensing data from SNs. Due to the advantages of low-cost, small size, flexibility and high mobility, UAVs have recently been employed in data collection for WSNs, which leads to the so-called UAV-assisted data collection network [14]. Furthermore, UAVs can fly near the wireless sensor nodes to achieve highly energy-efficient data transmissions over the line-of-sight (LoS) communication links, which is very helpful in lowering the energy consumption for SNs to transmit data, and hence extending the survival time of WSNs.

In UAV-assisted data collection networks, UAVs have found a substantial performance improvement with respect to effectiveness indicators, such as communication capacity and max-min rate, via jointly optimizing UAV trajectories and UAV-SN associations [15,16]. To further improve the spectral efficiency and support massive connectivity, NOMA is integrated with its user grouping and power allocation being optimized jointly to maximize the sum rate of a wireless sensor network [17]. The above works regarding UAV trajectory designs have assumed a fixed operation period for UAVs. In order to be compatible with time-sensitive services, the task completion time is minimized in cases of constrained energy [18] and NOMA-enabled [19]. In [20], the authors analytically characterize the optimal solution structure for the joint UAV trajectory design and SN scheduling. In [21], the energy budget of ground sensors is taken into account to further lower UAVs’ completion time. By taking the energy limitation of WSNs into consideration, a joint 3D trajectory design and data collection scheduling scheme is encouraged to save the energy of both UAVs and SNs [22], and the long-term energy consumption is minimized in [23]. Moreover, for large-scale IoT where a large amount of sensors are deployed, clustering sensors can significantly improve data acquisition efficiency. A cluster head selection scheme and the corresponding data forwarding rules within the cluster are proposed to maximize the value of information (VoI) [24]. Considering a multi-scenario parallel data collection task, the clustering strategy, cluster head mode selection, UAV flight trajectory and UAV velocity are jointly optimized to minimize the data collection time [25]. In [26], the clustering algorithm is improved to optimize the system’s energy efficiency. Besides, for applications that require real-time updates, such as connected vehicle networks, remote monitoring systems, etc., the AoI-minimal data collection is considered for UAV-assisted WPCNs [27] as well as UAV-aided IoT networks [28].

### 1.2. Contribution

It should be emphasized that all the aforementioned studies have focused on deterministic data collection tasks. In these scenarios, the locations of SNs and the fact that each SN has acquired valid data for collection are both predetermined and known in advance. However, In many IoT applications, such as wildlife monitoring and forest fire detection, ground sensors are sparsely distributed over vast areas. While these SNs continuously monitor their surroundings, they only intermittently capture meaningful data, like footage of wildlife activities, that require collection. In other words, the occurrence of SNs obtaining valuable data is probabilistic. Therefore, for a wide and sparse WSN, and considering the limited battery life on UAVs, maximizing the collection of valid data within the constraints of a UAV’s flight time presents a significant challenge. To the best of our knowledge, no studies have yet addressed the issue of indeterministic data collection.

To address the indeterministic data collection problem mentioned above, we first present a model of UAV-assisted wide and sparse wireless sensor network (WS-WSN), and formulate an effective data block sum maximization problem. Then, we develop a joint UAV trajectories design, SN serve scheduling and UAV-SN association algorithm is developed. Numerical results show that our proposed algorithm has better performance in the ratio of collected effective data blocks compared to baseline algorithms. The contributions presented in this paper are summarized as follows:

- We model a UAV-assisted wide and sparse wireless sensor network and formulate a novel indeterministic data collection problem. By our consideration, only a part of ground SNs obtain effective sensing data that contain target information. Under the wide and sparse wireless sensor network assumption, UAVs are not able to fly over and serve all of the SNs ergodically due to limited onboard energy. Accordingly, we formulate an effective data block sum maximization problem that aims to maximize the number of effective data blocks within a limited flying period.
- We propose a joint UAV trajectories design, SN serve scheduling and UAV-SN association algorithm. In particular, to deal with the indeterministic effectiveness indicator, we reformulate the problem by introducing an effective probability prediction model based on Deep Neural Network (DNN). Furthermore, to tackle the difficulty of varying constraints brought by partial data collection, a modified knapsack algorithm is improved.
- We provide numerical results to verify the performance of the proposed algorithm. We show that, compared to the non-effective prediction (NEP) scheme, the proposed scheme with effective probability prediction (EP) consumes much less time for collecting the same percent of effective data blocks. Besides, the proposed algorithm adopting EP based on DNN shows performance gain against the baseline algorithm adopting EP based on Random Forests (RF).

The remainder of this paper is organized as follows: In Section 2, we give the system model of WS-WSN and in Section 3 we formulate the effective data block sum maximization problem. In Section 4, we introduce effective probability prediction and reformulate the problem. In Section 5, we present the joint UAV trajectories design, SN serve scheduling and UAV-SN association algorithm. In Section 6, we illustrate numerical results and validate the performance of the proposed algorithm. Finally, In Section 7, we conclude the paper.

## 2. System Model

### 2.1. UAV-Assisted Wide and Sparse WSN

**Figure 1:**
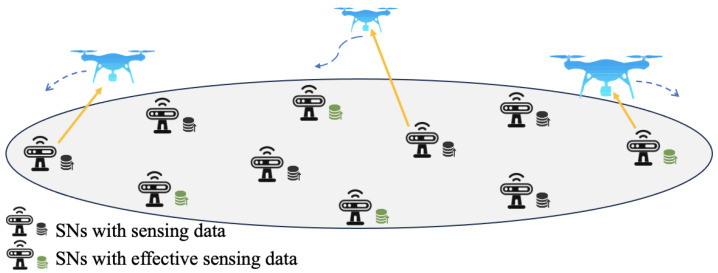
Indeterministic data collection in a UAV-assisted WS-WSN.

As shown in Figure 1, we consider a UAV-assisted wide and sparse distributed wireless sensor network (WS-WSN). M≥1 rotary-wing UAVs are employed to collect data from K≥1 SNs distributed on a ground area. Assume the ground area is wide enough such that SNs are sparsely distributed, i.e., the distance between two SNs is large enough that they can not be simultaneously covered by one UAV. Denote the set of UAVs and the set of SNs by M with |M|=M and K with |K|=K, respectively. The UAVs are assumed to fly at a fixed altitude *H* above the ground. Under a three-dimensional Cartesian coordinate system, the time-varying coordinate of UAV m∈M is denoted by $q_{m}\left(t\right)={\left[x_{m}\left(t\right),y_{m}\left(t\right),H\right]}^{T}\in R^{3\times 1}$. The exact location of SN k∈K is denoted by $s_{k}={\left[x_{k},y_{k},0\right]}^{T}\in R^{3\times 1}$, which is assumed to be fixed.

Assume that SNs sense the surrounding environment and produce sensing data periodically. Accordingly, the UAVs collect sensing data from SNs in a cycle mode, with the length of each flying period denoted as *T*. At the end of each period, UAVs return to depots for recharging and maintenance. Denote the locations of depots by $q_{m}\left(0\right)$ (m∈M). For ease of exposition, the period *T* is discretized into *N* equal time slots, with length $T_{s}=\frac{T}{N}$ chosen to be sufficiently small such that UAV locations are considered as approximately unchanged within each time slot even at the maximum flying speed $V_{\max}$. As a result, the flying trajectory of UAV *m* can be approximated by an *N*-length sequence $q_{m}\left[n\right]={\left[x_{m}\left[n\right],y_{m}\left[n\right],0\right]}^{T}$, n=1,⋯,N. During the *n*-th time slot, the distance between UAV *m* and SN *k* is $d_{m,k}\left[n\right]=\sqrt{∥q_{m}\left[n\right]-s_{k}{∥}^{2}+H^{2}}$.

### 2.2. Indeterministic Data Collection

During each period, each SN senses its surrounding environment and produces a data block of $D_{0}$ bits. In this paper, considering the monitoring applications, the sensing data are generally referred to multimedia data such as images and videos. Hence, $D_{0}$ usually takes a value which is much larger than the size of traditional simple sensing data such as temperatures and humidness. Only a part of the sensing data blocks which contain target information are effective. For example, in wildlife monitoring applications, only the data blocks containing information related with wildlife activities are effective. For the *l*-th sensing period, use a binary variable $e_{k}^{\left(l\right)}$ to denote the effectiveness of sensing data block at SN *k*. For low cost and low energy consumption purpose, assume that SNs have no computing ability and are incapable to judge the effectiveness of sensing data. It means that the data effectiveness is indeterministic until it is collected and processed by a UAV. Besides, it is reasonable to assume that the number of UAVs is far smaller than the number of SNs, i.e., M≪K. Thus, UAVs do not have enough time and energy to fly over and serve all of the SNs ergodically within a UAV flying period *T*.

### 2.3. Average Data Collection Rate

For the *l*-th flying period, define a set of binary variables $\left\{\alpha_{m,k}^{\left(l\right)}\left[n\right]\right\}$ to represent the association relationship between UAVs and SNs. $\alpha_{m,k}^{\left(l\right)}\left[n\right]=1$ indicates that SN *k* is served by UAV *m* in the *n*-th time slot of the *l*-th flying period, otherwise $\alpha_{m,k}\left[n\right]=0$. Under the wide and sparse WSN assumption, to facilitate the cooperation among multiple UAVs in order to cover more SNs, we assume that within a time slot, each SN is only served by at most one UAV, and a UAV serves no more than one SN. By this assumption, a simple communication protocol can satisfy the communication needs. There are many classic protocols that can be used, such as UAVCAN, IEEE 802.11, etc. The specific protocol design and implementation are not within the scope of this paper. Thus, we have two association constraints expressed as

(1) $$\begin{array}{cc} \sum_{m=1}^{M}\alpha_{m,k}^{\left(l\right)}\left[n\right] & \leq 1,\;\forall l,n,k, \end{array}$$

(2) $$\begin{array}{cc} \sum_{k=1}^{K}\alpha_{m,k}^{\left(l\right)}\left[n\right] & \leq 1,\;\forall l,n,m. \end{array}$$

Besides, considering that not all SNs are served by the UAV network, define a set of binary variables $\left\{\beta_{k}\right\}$ to represent whether an SN is served by UAVs during flying period *l*. Obviously, the association variables $\left\{\alpha_{m,k}^{\left(l\right)}\left[n\right]\right\}$ and the serve scheduling variables $\left\{\beta_{k}\right\}$ should satisfy

(3) $$\begin{array}{c} \sum_{n=1}^{N}\sum_{m=1}^{M}\alpha_{m,k}^{\left(l\right)}\left[n\right]\geq \beta_{k}^{\left(l\right)},\;\forall k. \end{array}$$

We assume that the air-to-ground channels between UAVs and SNs are dominated by line-of-sight (LoS) channels [22]; in this paper, the NLoS components have limited impact on the transmission between the UAVs and SNs, and can be ignored for two reasons. First, in this paper, we consider the case that SNs being deployed in a wide and open area, such as farmland, or animal ecotope. With the minimum flying height limitation for UAVs, the probability of NLoS is relatively small. Second, the UAVs tend to fly to the locations above each SN in turn for better communication performance, making the probability of LoS larger. During time slot *n*, the channel power gain from UAV *m* to SN *k* is given by $h_{m,k}\left[n\right]=\rho_{0}d_{m,k}^{-2}\left[n\right]=\frac{\rho_{0}}{∥q_{m}\left[n\right]-s_{k}{∥}^{2}+H^{2}}$, where $\rho_{0}$ is the channel power gain at a reference distance of 1 m (m). The received signal-to-interference-plus-noise ratio (SINR) at UAV *m* can be expressed as

(4) $$\begin{array}{c} \gamma_{m,k}\left[n\right]=\frac{P_{s}h_{m,k}\left[n\right]}{\sum_{k^{\prime }=1,k^{\prime }\neq k}^{K}P_{s}h_{m,k^{\prime }}\left[n\right]+\sigma^{2}}, \end{array}$$

where $P_{s}$ and $\sigma^{2}$ denote the transmit power of SNs and the Gaussian noise term, respectively. We assume that the UAVs collect data from SNs through the same time-frequency channel. Thus, a UAV receiver may experience interference from other SN-UAV transmission, with the interference power denoted as $\sum_{k^{\prime }=1,k^{\prime }\neq k}^{K}P_{s}h_{m,k^{\prime }}\left[n\right]$. The data transmission rate from SN *k* to UAV *m* in the time slot *n* in bits/second/Hertz (bps/Hz) is

(5) $$\begin{array}{c} R_{m,k}\left[n\right]=\log_{2}\left(1+\gamma_{m,k}\left[n\right]\right). \end{array}$$

The average data collection rate at SN *k* over the *N* time slots of a flying period is given by

$$\begin{array}{cc} R_{k} & =\frac{1}{N}\sum_{n=1}^{N}\sum_{m=1}^{M}\alpha_{m,k}^{\left(l\right)}\left[n\right]R_{m,k}\left[n\right]. \end{array}$$

## 3. Effective Data Block Sum Maximization

For a wide and sparse WSN, UAVs are unable to serve all of the SNs within a flying period due to the energy limitation. We expect the UAV to collect as many effective data blocks as possible. Hence, we formulate a problem that maximizes the sum of effective sensing data blocks via jointly optimizing the UAV trajectories, SN serve scheduling as well as UAV-SN associations, formulated by

(6a) $$\begin{array}{cc} \left(P1\right): & \max_{Q,A,B}\;\sum_{k=1}^{K}e_{k}^{\left(l\right)}\beta_{k}^{\left(l\right)}\\ s.t.\;\;\;\; & (1),(2),(3), \end{array}$$

(6b) $$\begin{array}{cc} & \alpha_{m,k}^{\left(l\right)}\left[n\right]\in \left\{0,1\right\},\;\forall m,k,n, \end{array}$$

(6c) $$\begin{array}{cc} & \beta_{k}^{\left(l\right)}\in \left\{0,1\right\},\;\forall k, \end{array}$$

(6d) $$\begin{array}{cc} & NR_{k}\geq D_{0},\;\forall k\in \left\{K\;|\;\beta_{k}^{\left(l\right)}=1\right\}, \end{array}$$

(6e) $$\begin{array}{cc} & ∥q_{m}\left[n\right]-q_{m}\left[n-1\right]∥\leq \min\left(T_{s}V_{\max},\Delta_{\max}\right),\;\forall n,\forall m, \end{array}$$

(6f) $$\begin{array}{cc} & ∥q_{m}\left[n\right]-q_{j}\left[n\right]∥\geq d_{\mathrm{safe}},\;\;\forall n,\forall m\neq j. \end{array}$$

where $Q=\left\{q_{m}\left[n\right],\forall m,n\right\}$, $A=\left\{\alpha_{m,k}^{\left(l\right)}\left[n\right],\forall m,k,n\right\}$ and $B=\{\beta_{k}^{\left(l\right)},\forall k\}$ are variables of UAV trajectories, UAV-SN associations, and SN serve scheduling, respectively. The object function $\sum_{k=1}^{K}e_{k}^{\left(l\right)}\beta_{k}^{\left(l\right)}$ denotes the total number of effective sensing data blocks collected by UAVs within the *l*-th flying period. Constraint (6d) guarantees that for each SN served by UAVs, i.e., SN $k\in \{K\;|\;\beta_{k}=1\}$, the data block of $D_{0}$ bits can be completely collected by UAVs within a flying period. Constraint (6e) restricts both the UAV speed and the finite-sum approximation error introduced by the time discretization for UAV trajectories, with $V_{\max}$ and $\Delta_{\max}$ denoting the maximum UAV speed and the maximum discretization segment length, respectively. Constraint (6f) ensures collision avoidance between different UAVs with $d_{\mathrm{safe}}$ denoting the minimum inter-UAV distance. Problem (P1) is difficult to tackle since the data effectiveness indicators $e_{k}^{\left(l\right)}$$\left(k\in K\right)$ contained in the objective function are indeterministic.

## 4. Effective Probability Prediction and Problem Reformulation

To make problem (P1) tractable, we first tackle the indeterministic indicators $e_{k}^{\left(l\right)}$. We predict the data’s effective probability by a deep learning-based model, and reformulate (P1) into an effective probability-weighted data block sum maximization problem, the parameters of which are determinate.

### 4.1. Data Effective Probability Prediction

Define the effective probability of sensing data at SN *k* during the *l*-th flying period as

(7) $$\begin{array}{c} p_{k}^{\left(l\right)}=P\left(e_{k}^{\left(l\right)}=1\right). \end{array}$$

Use $P^{\left(l\right)}={\left\{p_{k}^{\left(l\right)}\right\}}_{k=1}^{K}$ to denote the effective probability vector of all SNs. Considering that in essence, $p_{k}^{\left(l\right)}$ is the probability that an event occurs. For example, in fire monitoring, $p_{k}^{\left(l\right)}=1$ is the probability of a fire happening near SN *k* during period *l*. In wildlife monitoring, $p_{k}^{\left(l\right)}=1$ is the probability of animals passing by SN *k* during period *l*. Hence, the values of $P^{\left(l\right)}$ are highly relative to the features related to the event and can be predicted by relevant feature data.

We construct an effective probability prediction model, aiming to approximate a function mapping φ:X→Y, where *X* is a set of input variables including the feature data of SNs such as position coordinates, temperature, humidness, etc. *Y* is the output variable denoting the effective probability of sensing data at SNs. It should be specifically noted that the environmental perception data mentioned above are relatively small in volume, typically several tens to several hundred bytes, and can be quickly and easily obtained within a few minutes before the formal sensing data collection process begins. For example, a UAV can be dispatched to fly along a circular trajectory that covers the area to collect the environmental perception data. For a 0.5×0.5 ${\mathrm{km}}^{2}$ square area, it only takes 40 s to fly around the area when the UAV is flying at a speed of 50 m/s. After collecting the environmental perception data, the UAV returns to the control center. Then, the control center would predict the effectiveness of the sensing data and plan the UAVs’ trajectories for sensing data collection. Compared to the environmental perception data, the sensing data used for detection and recognition tasks, such as surveillance video data, are relatively larger in volume, reaching gigabytes (Gb). UAVs are required to fly close to the SNs and hover for a long period to collect sensing data. It is inefficient and excessively energy-consuming for UAVs to collect sensing data from all of the SNs. Therefore, we need to predict the effectiveness of sensing data based on the environmental perception data and optimize the collection efficiency.

A deep learning-based modeling framework is adopted. The training dataset is

(8) $$\begin{array}{c} T_{I}=\left\{\left(x_{k,i},y_{k,i}:k\in K,i\in \left[1,I\right]\right)\right\}, \end{array}$$

where *I* is the number of records in the training dataset, $x_{k,i}$ and $y_{k,i}$ are, respectively, the training inputs and target output of the *i*-th record related to SN *k*. The training determines the parameters of the model that minimize the loss function between the estimated and real values of the output variables, yielding a non-linear interpolation-based input-output mapping φ. Hence, the matrix $P^{\left(l\right)}$ can be predicted by

(9) $$\begin{array}{c} {\hat{P}}^{\left(l\right)}=\varphi \left(X^{\left(l\right)}\right). \end{array}$$

### 4.2. Problem Reformulation

By substituting the effectiveness indicators $e_{k}^{\left(l\right)}$ with the predicted effective probability $p_{k}^{\left(l\right)}$, problem (P1) can be reformulated into an expected effective data block sum maximization problem (EE-SMP), which aims to maximize the expected sum of effective data blocks, given by

(10) $$\begin{array}{cc} \left(P2\right): & \max_{Q,A,B}\;\sum_{k=1}^{K}p_{k}\beta_{k}\\ s.t.\;\;\;\; & (1),(2),(3),(6b),(6c),(6d),(6e),(6f). \end{array}$$

The subscript *l* is omitted.

Solving the reformulated problem (P2) is still challenging, since (P2) is quite different from traditional joint UAV trajectories design and UAV-SN association optimization problems. In particular, the number of constraints in (P1) given by (6d) varies with its optimization variable, i.e., the UAV-SN scheduling indicator $B=\{\beta_{k}^{\left(l\right)},\forall k\}$, since (6d) only restricts the SNs that are served by UAVs.

## 5. Modified Knapsack Algorithm

The EE-SMP given by (P2) is a complicate combinational optimization problem. Besides, (P2) contains varying constraints, i.e., the constraint given by (6d) varies with the specific value of the serve scheduling variables $B=\{\beta_{k},\forall k\}$. It means that, if an SN *k* is chosen to be served by UAV, i.e., $\beta_{k}=1$, then the lower bound of its average transmission rate $R_{k}$ should be limited to ensure the collection of a complete data block. Otherwise, for a non-served SN, there is no minimum data collection rate limitation. Hence, problem (P2) can not be treated as a traditional optimization problem.

We solve the EE-SMP by modeling it as a modified knapsack problem. First, we treat each sensor node, named SN *k*, as an item, with its data effective probability $p_{k}$ being viewed as the value of the item. Then, we create a virtual knapsack to accommodate the items. Maximizing the expected effective data block sum $\sum_{k=1}^{K}p_{k}\beta_{k}$ via optimizing the serve scheduling strategy $B=\{\beta_{k},\forall k\}$, as given in (P2), is equivalent to finding a packing strategy B that maximizes the total value of the virtual knapsack. Hence, the EE-SMP can be equivalently reformulated into a modified knapsack problem, given by

(11a) $$\begin{array}{cc} \left(P3\right): & \max_{\tilde{B}}\;\sum_{k=1}^{K}p_{k}{\tilde{\beta }}_{k} \end{array}$$

(11b) $$\begin{array}{cc} s.t.\;\;\; & {\tilde{\beta }}_{k}\in \left\{0,1\right\},\;\forall k, \end{array}$$

(11c) $$\begin{array}{cc} & {\left(P2\right)|}_{B=\tilde{B}}\;\mathrm{is}\;\mathrm{feasible}. \end{array}$$

(11c) means that the packing strategy $\tilde{B}=\left\{\beta_{k},\forall k\right\}$ should be feasible to (P2) in order to make it solvable. (P3) is a modified knapsack problem (MKP) with the capacity constraint in traditional knapsack problem being replaced with a feasibility constraint given by (11c). To solve (P3), we first solve the feasibility problem of (P2) with given B, i.e., ${\left(P2\right)|}_{B=\tilde{B}}$, and then we resort to the greedy algorithm to solve the MKP.

### 5.1. The Feasibility Problem of ${\left(P2\right)|}_{B=\tilde{B}}$

Given $B=\tilde{B}$, the feasibility problem of (P2) can be expressed as

(12a) $$\begin{array}{cc} \left(P4\right): & \;\mathrm{find}\;\;\;Q,A \end{array}$$

(12b) $$\begin{array}{cc} s.t.\;\;\;\; & \sum_{n=1}^{N}\sum_{m=1}^{M}\alpha_{m,k}\left[n\right]\geq {\tilde{\beta }}_{k},\;\forall k, \end{array}$$

(12c) $$\begin{array}{cc} & NR_{k}\geq D_{0},\;\forall k\in \left\{K\;|\;{\tilde{\beta }}_{k}=1\right\}, \end{array}$$

(12d) $$\begin{array}{cc} \; & (1),(2),(6e),(6f). \end{array}$$

(P4) can be further transformed into a max-min rate problem given by

(13a) $$\begin{array}{cc} \left(P5\right): & \max_{\tilde{A},Q,\eta }\;\eta \end{array}$$

(13b) $$\begin{array}{cc} s.t.\;\;\;\; & NR_{k}\geq \eta ,\;\forall k\in \tilde{K}, \end{array}$$

(13c) $$\begin{array}{cc} \; & (1),(2),(6e),(6f). \end{array}$$

where $\tilde{A}=\left\{A|\sum_{n=1}^{N}\sum_{m=1}^{M}\alpha_{m,k}\left[n\right]\geq {\tilde{\beta }}_{k}\right\}$ and $\tilde{K}=\left\{K\;|\;{\tilde{\beta }}_{k}=1\right\}$ are the valid UAV-SN association variables and the set of served SNs given $B=\tilde{B}$, respectively. (P4) is feasible if and only if the optimal object value of (P5), denoted by $\eta^{\mathrm{opt}}$, satisfying $\eta^{\mathrm{opt}}\geq D_{0}$. (P5) is a classic joint UAV trajectory and UAV-SN associations optimization problem. We decompose it into two sub-problems, and resort to the block coordinate decent (BCD) and successive convex approximation (SCA) techniques. The two sub-problems are alternately solved until the algorithm converges, as referred in [29].

### 5.2. Algorithm Design

Based on the solution to the feasibility problem of (P2), a hybrid greedy algorithm is proposed to resolve the modified knapsack algorithm, as summarized in Algorithm 1.
**Algorithm 1** Hybrid greedy algorithm for (P3)1: **repeat**2:   Initialize iteration index r=0, set item buffer $I^{0}=K$, set knapsack pack $B^{0}=0$ and knapsack value $v^{0}=0$.3:   For the *i*-th iteration, find the item $k^{r}$ which has the maximum value in $I^{r}$. Take $k^{r}$ out from $I^{r}$, and set $I^{r+1}=I^{r}-k^{r}$.4:   Set $\tilde{B}=B^{r}$.5:   Update $\tilde{B}$ by setting ${\tilde{\beta }}_{k^{r}}=1$.6:   Check the feasibility of ${\left(P2\right)|}_{B=\tilde{B}}$ via solving (P5).7:   **if** ${\left(P2\right)|}_{B=\tilde{B}}$ is feasible **then**8:     Put item $k^{r}$ into knapsack with updating $B^{r+1}=\tilde{B}$.9:   **else**10:     Discard item $k^{r}$ with updating $B^{r+1}=B^{r}$.11:     Go back to step 3.12:   **end if**13:   update r=r+1.14: **until** $I^{r+1}$ is empty.15: Output $B^{r+1}$ as the optimal knapsack packing strategy.

## 6. Numerical Results

In this section, we provide numerical results to demonstrate the effectiveness of the proposed scheme. We consider a scenario where the sensing nodes are sparsely distributed, where K=20 SNs are randomly and uniformly distributed within a 2D square area of 0.5×0.5 ${\mathrm{km}}^{2}$. Two UAVs are employed to collect data from ground SNs. For the effective probability prediction model training, a natural area sensing dataset from a Chinese telecom operator is adopted. As part of research collaborative efforts, we were granted access to the labeled data collected by 20 sensors monitoring a forestry area in 2022. The total number of sensing records is 24,612. For each sensing record, the input variables include position coordinates, altitudes, temperature, humidness, wind strength, pressure, light, and sound volume, and the output is a Boolean variable indicating the data effectiveness. A two-layer DNN network is used to train the prediction model;compared to the traditional machine learning algorithms like decision trees [30] and linear models [31], DNN is distinguished by deep, hierarchical architecture, which facilitates the learning of complex, nonlinear patterns within data. When dealing with predictive tasks, DNNs often demonstrate superior predictive performance. The main simulation setups are summarized in Table 1.

Two baseline schemes are adopted for comparison. (1) MKP with effective probability predicted by Random Forests (EP-RF). (2) UAVs fly along a Traveling Salesman route without effective probability prediction (NEP). In particular, UAVs determine whether to fly to the nearest SN to collect data, or fly back to depots to recharge, according to remaining battery energy.

### 6.1. DNN-Based Effective Probability Prediction

The variables of each record for model training include position coordinates $\left(X_{1},X_{2}\right)$, altitudes $\left(X_{3}\right)$, temperature $\left(X_{4}\right)$, humidness $\left(X_{5}\right)$, wind strength $\left(X_{6}\right)$, pressure $\left(X_{7}\right)$, light $\left(X_{8}\right)$, “sound volume” $\left(X_{9}\right)$ and a Boolean variable indicating whether each SN obtains an effective sensing data block (Y). We normalize all continuous variables by Z-Score normalization, formulated as

(14) $$\begin{array}{c} {\tilde{x}}_{i}^{\left(j\right)}=\left(x_{i}^{\left(j\right)}-\mu_{i}\right)/\sigma_{i},\;\forall i,j. \end{array}$$

where $x_{i}^{\left(j\right)}$ is the value of variable $X_{i}$ of the *j*-th record, $\mu_{i}$ and $\sigma_{i}$ are the mean and the standard deviation of $X_{i}$, respectively. The dataset has been used for training and testing in a 90:10 ratio.

**Figure 2:**
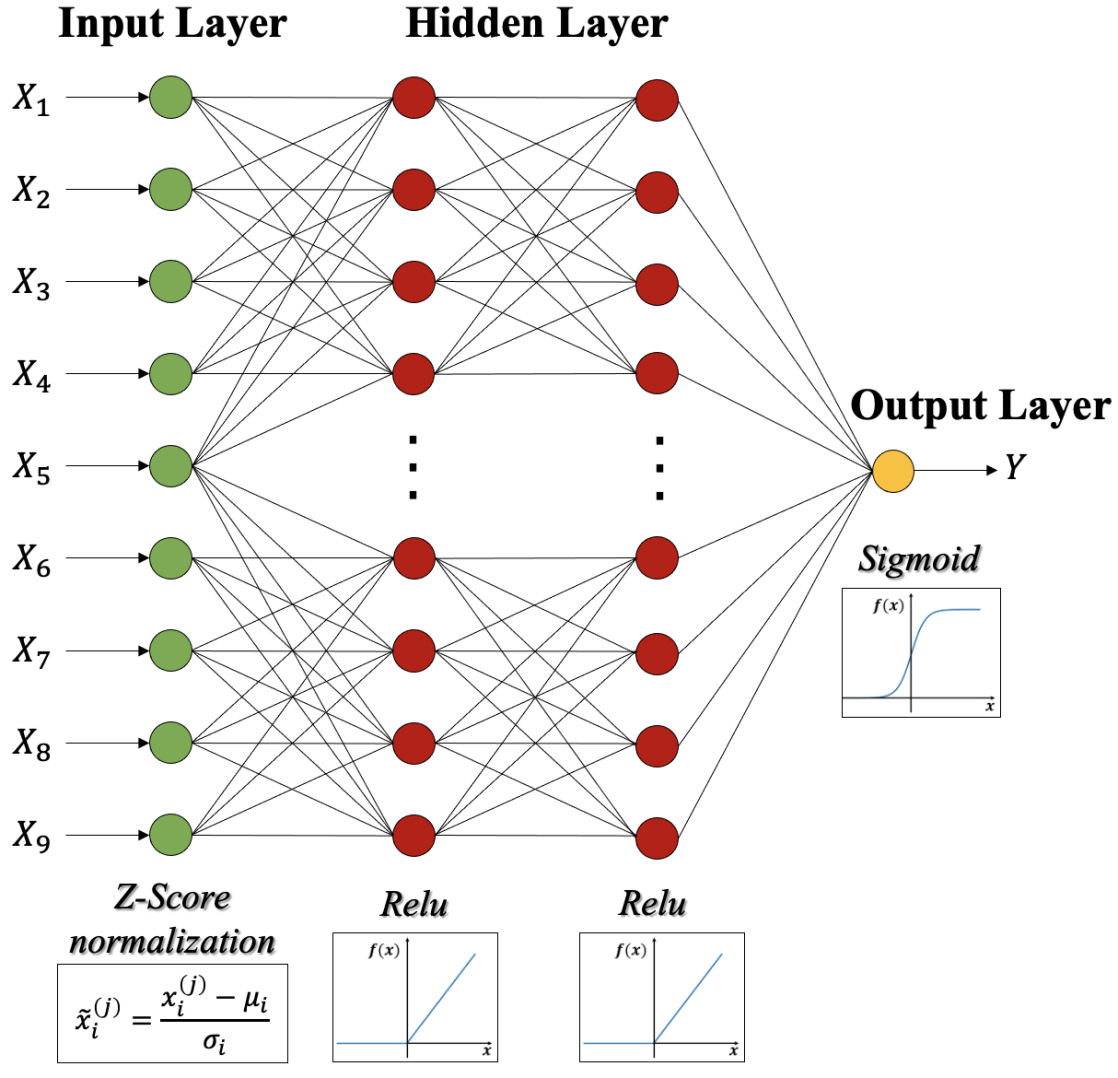
The DNN network of the data effective probability prediction model.

A DNN network has been used to train the prediction model, which contains an input layer, two hidden layers and an output layer, as shown in Figure 2. We use normalized data $X_{1},X_{2},\cdots ,X_{9}$ as the inputs of the DNN network and *Y* as the output. Through repeated testing and verification, it was found that the two hidden layers with 50 neurons in each layer were able to provide enough nonlinear expression ability. We use the ReLU function to process the input of the hidden layer neurons, and the Sigmoid function to process the output.

**Figure 3:**
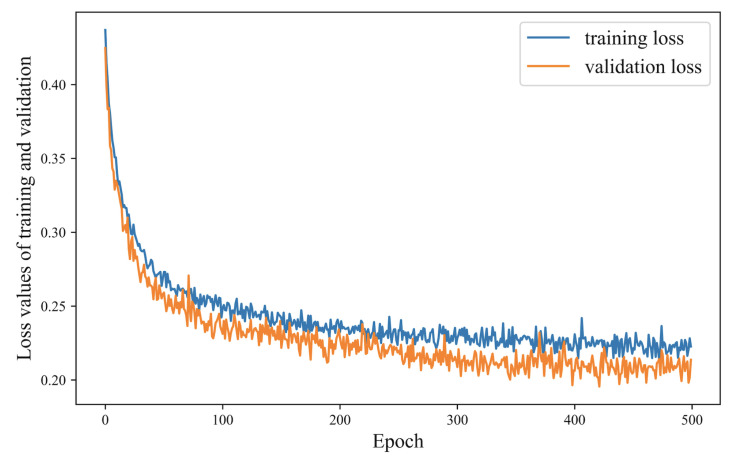
The loss value of training and validation at successive epochs.

**Figure 4:**
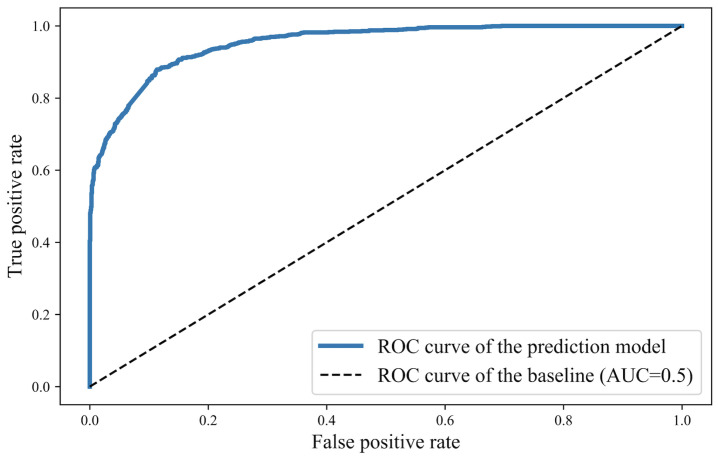
The ROC curve of performance evaluation on test dataset.

We conduct training and performance tests on Intel Core i7-8569 and 16G RAM. The number of epochs and batch size are set as 500 and 128, respectively. The Adam optimizer with a learning rate of 0.01 is used. Cross entropy is adopted as the loss function, and 10% of the training data have been used as a validation set, which has been used for model selection and hyperparameter optimization. The validation set has provided an unbiased evaluation of a model fit during the training phase as it was not used in the training process itself. The loss value of training and validation at successive epochs is shown in Figure 3. When the model training has been completed, the test set is used to evaluate the final performance of the model, which has been completely unseen by the model during both the training and validation phases. We evaluated the prediction model by ROC curve, and performance evaluation on the test set is shown in Figure 4. The area under the curve (AUC) is 0.953.

### 6.2. Illustrations of Optimized UAV Trajectories and SN Serve Scheduling

**Figure 5:**
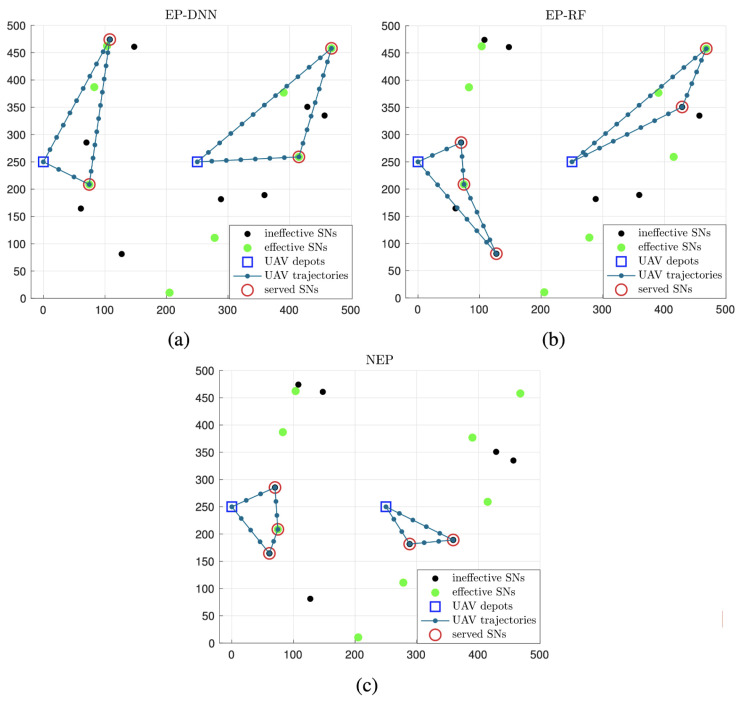
The optimized UAV trajectories and SN serve scheduling of the EP-DNN scheme (**a**), the EP-RF scheme (**b**) and the NEP scheme (**c**) when T=40 s.

**Figure 6:**
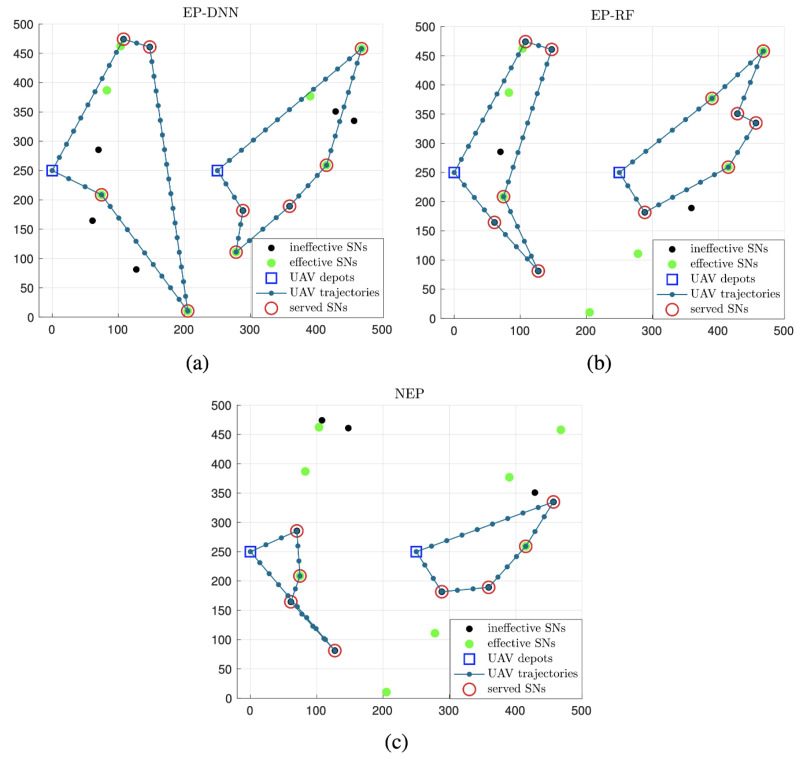
The optimized UAV trajectories and SN serve scheduling of the EP-DNN scheme (**a**), the EP-RF scheme (**b**) and the NEP scheme (**c**) when T=80 s.

Figure 5 and Figure 6 give typical results of optimized UAV trajectories and SN serve scheduling with the three schemes in one implementation, when the UAV flying period *T* set to be 40 s and 80 s, respectively. It is observed that, with the two schemes with effective probability prediction, i.e., EP-DNN and EP-RF, UAVs tend to choose SNs of greater predicted effective probability, which may be located far away from the depots. Moreover, among the two EP-based schemes, UAVs with the EP-DNN scheme show much more accuracy via choosing more effective SNs, since DNN performs better than RF in prediction accuracy. In contrast, with the NEP scheme, UAVs give preference to the nearby SNs around the depots, and collect more ineffective data than the other two schemes. Comparing Figure 5 and Figure 6, it shows that with a larger flying period *T*, the EP-based schemes almost cover all of the effective SNs. While with the NEP scheme, a high proportion of sensing data are collected from ineffective SNs.

### 6.3. Collected Data Block Ratios

**Figure 7:**
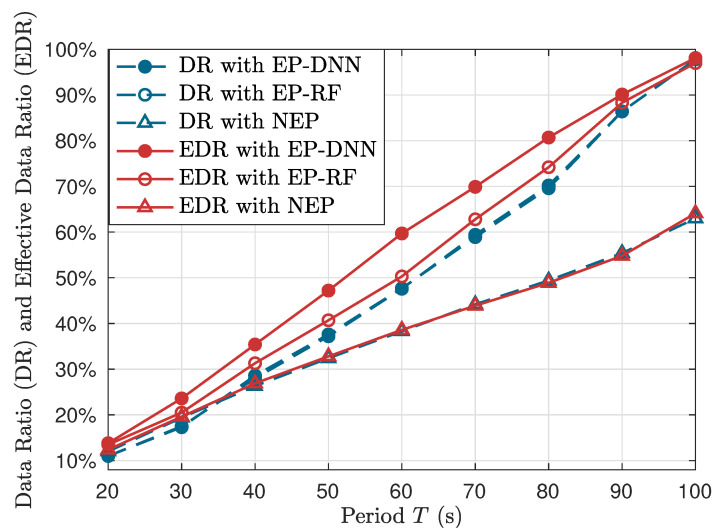
The collection ratio of data blocks (DR) and effective data blocks (EDR) with respect to the flying period *T*.

To compare the performance of different schemes, we consider two metrics regarding data collection performance. One is the collection ratio of all data blocks (DR), i.e., the ratio of the number of collected data blocks and the total number of data blocks. The other is the collection ratio of effective data blocks (EDR), i.e., the ratio of the number of collected effective data blocks and the total number of effective data blocks. Figure 7 shows the curves of DR (plotted by blue and dotted curves) and EDR (plotted by red and solid curves) with respect to the flying period *T*. When *T* is small, the EP-DNN, EP-RF and NEP schemes show similar performance in both DR and EDR, since the period time is only enough for UAVs flying around depots. As *T* increases, the two EP-based schemes, exhibit advantages in both DR and EDR against the NEP scheme, with the performance gaps also increasing. In particular, the DR performance of EP-DNN and EP-RF is similar, with the gain compared to NEP coming from joint trajectories and serve scheduling optimization. While the EDR performance of EP-DNN is better than EP-RF, due to the prediction accuracy gain of EP-DNN against EP-RF. When *T* is large enough, e.g., taking values of 90 s or 100 s, the performance curves of EP-DNN and EP-RF converges, since in this case, UAVs are able to serve almost all of the SNs. For a quantitative comparison, consider 50% effective data collection, the NEP scheme takes near 80 s, while the EP-DNN and EP-RF schemes take 52 s and 60 s, corresponding to near 35% and 25% time reduction, respectively.

**Figure 8:**
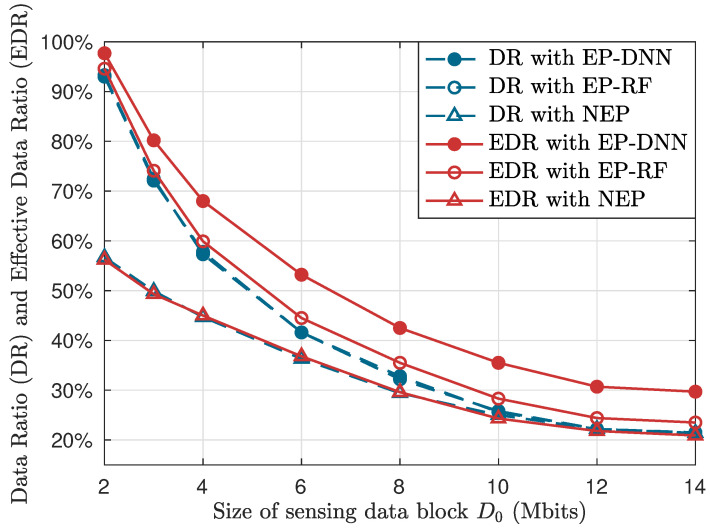
DR and EDR with respect to the data block size $D_{0}$, with *T* set as 80 s.

Figure 8 shows the curves of DR (plotted by blue and dotted curves) and EDR (plotted by red and solid curves) with respect to the data block size $D_{0}$ given T=80 s. When $D_{0}$ is small, UAVs consume less time in data collection from SNs and remain more time for UAVs to fly and visit more SNs. In this case, the EP-DNN scheme and the EP-RF scheme show obvious performance gains in both DR and EDR compared to the NEP scheme. With $D_{0}$ increases, it is observed that the advantages of the two EP schemes reduce, since the time remaining for UAVs to fly decreases, and hence the space for trajectory optimization decreases. Besides, it is worth noticing that, as $D_{0}$ increases, the gap between EP-DNN and EP-RF becomes first large and then stable. The reason is that, when $D_{0}$ is small, UAVs are able to visit more SNs, and probabilistically include more effective SNs, hence the prediction accuracy becomes less important.

## 7. Conclusions

In this paper, we focus on an indeterministic data collection task for UAV-assisted wide and sparse wireless sensor networks, where the SNs obtain effective data randomly, and the UAV has no pre-knowledge about which sensor has effective data. We jointly optimized the UAV trajectories, SN serve scheduling and UAV-SN associations to maximize the sum of collected effective sensing data blocks. We addressed the indeterministic effective indicator by introducing an effectiveness probability prediction model and tackled the issue caused by a varying number of constraints by proposing a two-layer modified knapsack algorithm, within which a feasibility problem is resolved iteratively to find the optimal packing strategy. Numerical results demonstrate that the proposed scheme had remarkable advantages in the sum of effective data blocks, and reduced the completion time for collecting the same ratio of effective data by nearly 30%.

## Figures and Tables

**Table 1:** Simulation parameters.

Parameter	Description	Value
*T*	Flying period of UAV	20–100 s
$D_{0}$	Sensing data block size	2–14 Mbits
$\rho_{0}$	Channel power gain at the reference distance 1 m	−30 dB
$\sigma^{2}$	Receiver noise power	−110 dBm
$\Delta_{\max}$	Maximum discretization segment length	5 m
$V_{\max}$	Maximum UAV speed	20 m/s
$D_{\mathrm{safe}}$	Minimum inter-UAV distance to ensure collision avoidance	50 m
$T_{s}$	Length of discretized time slot	1 s
$P_{s}$	Transmit power of SN	5 mW
*H*	Flying altitude of UAVs	80 m

## Data Availability

Data are contained within the article.
